# Realistic precision and accuracy of online experiment platforms, web browsers, and devices

**DOI:** 10.3758/s13428-020-01501-5

**Published:** 2020-11-02

**Authors:** Alexander Anwyl-Irvine, Edwin S. Dalmaijer, Nick Hodges, Jo K. Evershed

**Affiliations:** 1 MRC Cognition and Brain Sciences Unit, University of Cambridge, Cambridge, UK; 2 Cauldron Science, St Johns Innovation Centre, Cambridge, UK

**Keywords:** Accuracy, Experiment builder, Big data, Reaction time, MTurk, Online testing, System testing, Automated hardware testing, Psychophysics

## Abstract

Due to increasing ease of use and ability to quickly collect large samples, online behavioural research is currently booming. With this popularity, it is important that researchers are aware of who online participants are, and what devices and software they use to access experiments. While it is somewhat obvious that these factors can impact data quality, the magnitude of the problem remains unclear. To understand how these characteristics impact experiment presentation and data quality, we performed a battery of automated tests on a number of realistic set-ups. We investigated how different web-building platforms (Gorilla v.20190828, jsPsych v6.0.5, Lab.js v19.1.0, and psychoJS/PsychoPy3 v3.1.5), browsers (Chrome, Edge, Firefox, and Safari), and operating systems (macOS and Windows 10) impact display time across 30 different frame durations for each software combination. We then employed a robot actuator in realistic set-ups to measure response recording across the aforementioned platforms, and between different keyboard types (desktop and integrated laptop). Finally, we analysed data from over 200,000 participants on their demographics, technology, and software to provide context to our findings. We found that modern web platforms provide reasonable accuracy and precision for display duration and manual response time, and that no single platform stands out as the best in all features and conditions. In addition, our online participant analysis shows what equipment they are likely to use.

## Introduction

**Fig. 1:**
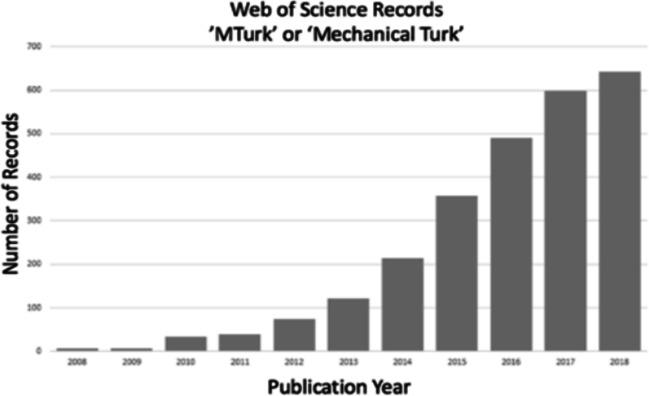
Trends over time in papers mentioning Mechanical Turk, taken from Web of Science

Conducting behavioural research online has vastly increased in the past few years. For instance, the number of papers tracked by Web of Science with the keywords ‘MTurk’ or ‘Mechanical Turk’ (Amazon’s popular platform for accessing online participants or workers, available since 2005) was 642 in 2018, over a five-fold increase over five years from 121 publications in 2013 (Fig. 1). While scientists do not exclusively use MTurk for psychological experiments, it is indicative of a trend. For example, Bohannon (2016) reported that published MTurk studies in social science increased from 61 in 2011 to 1200 in 2015—an almost 20-fold increase.

A unique problem with internet-based testing is its reliance on participants’ hardware and software. Researchers who are used to lab-based testing will be intimately familiar with their computer, stimulus software, and hardware for response collection. At the very least, they can be sure that all participants are tested using the very same system. For online testing, the exact opposite is true: participants use their own computer (desktop, laptop, tablet, or even phone), with their own operating system, and access experiments through a variety of web browsers.

In addition to participant degrees of freedom, researchers can choose between various options to generate experiments. These vary from programming libraries (e.g. jsPsych) to graphical experiment builders (e.g. Gorilla Experiment Builder), and come with their own idiosyncrasies with respect to timing, presentation of visual and auditory stimuli, and response collection.

This presents a potential problem for researchers: Are all of the unique combinations of hardware and software equal? Here, we first investigate the types of software that potential participants use, and how common each option is. We then provide a thorough comparison of the timing precision and accuracy of the most popular platforms, operating systems, internet browsers, and common hardware. We specifically compare four frequently used platforms that facilitate internet-based behavioural research:

- Gorilla Experiment Builder build 20190828 (www.gorilla.sc)
- jsPsych v6.0.5 (www.jspsych.org)
- Lab.js v19.1.0 (lab.js.org)
- PsychoJS v3.1.5 (building in PsychoPy3, and hosting on www.pavlovia.org)

We included these packages because they are among the most frequently used platforms, in our experience, but little quantitative data is available to support this. Regrettably, other notable platforms such as LabVanced (www.labvanced.com) and the OSWeb extension to OpenSesame (Mathôt, Schreij, & Theeuwes, 2012) have remained untested here due to practical restrictions on our time and resources.

### A brief history of online experiments

The almost exponential increase in papers citing MTurk is surprisingly recent. While the internet has been available since the 1990s, and tools like MTurk have existed since the mid-2000s, the adoption of online research has begun to accelerate only in the past 5–10 years. There are, however, some early examples of online experimentation, for example, investigating spatial cognition (Givaty et al., 1998), visual motion extrapolation (Hecht et al., 1999), probability learning (Birnbaum & Wakcher, 2002), and establishment of labs dedicated to web experiments (Reips, 2001). In the late 1990s and early 2000s, several guidance books and articles on the subject were published (Birnbaum, 2000; McGraw et al., 2000), with one 1995 review even coining the term ‘Cyberpsych’ to describe internet-based psychological science (Kelley-Milburn & Milburn, 1995). Sadly, it appears that the term did not catch on. Articles providing technical guidance published for running experiments, such as maintaining a web server (Schmidt et al., 1997) and analysing server logs (Reips & Stieger, 2004), also emerged around this time. However, despite the availability of these tools and the promise of larger sample sizes, it took years to reach the current high levels of demand. There are several potential explanations for this apparent research adoption lag: the required level of technical ability, availability of personal devices, and concerns over data quality.

Building a research project online in the late 2000s required a much higher level of web-specific technical skills. Experimenters would have to have known how to construct web pages and load resources (e.g. images and videos), capture and transmit participant data, configure and maintain a server to host the web pages and receive the participant data, and store the participant data in a database. Additionally, the capabilities of web applications at this time did not allow for much more than slow image and text presentation. Interactive animations and dynamic elements were inconsistent, and often slow to load for most users. There were survey tools available such as Qualtrics, Survey Monkey, and Lime Survey (Baker, 2013), but these really only permitted relatively simple experiments.

In the early 2010s, the situation began to change with better tools becoming available. In particular, the High Resolution Time API, which allowed for far better timing accuracy than older methods such as setTimeout(), began appearing in browsers in 2013 (although it was not supported in all major browsers until 2015—www.caniuse.com/#feat=high-resolution-time). Running online research, allowing dynamic presentation of experimental trials and stimuli, and recording reaction times was possible through tools such as QRTEngine (Qualtrics Reaction Time Engine; Barnhoorn, Haasnoot, Bocanegra, & Steenbergen, 2015) and jsPsych v6.0.5 (JavaScript Library for building and presenting experiments; de Leeuw, 2015), which originally appeared around 2013. As more tools and platforms have become available (for an overview, see Anwyl-Irvine, Massonnié, Flitton, Kirkham, & Evershed, 2019), the technical barrier to web-based research seems to have been at least partially alleviated, allowing more research to be conducted online.

**Fig. 2:**
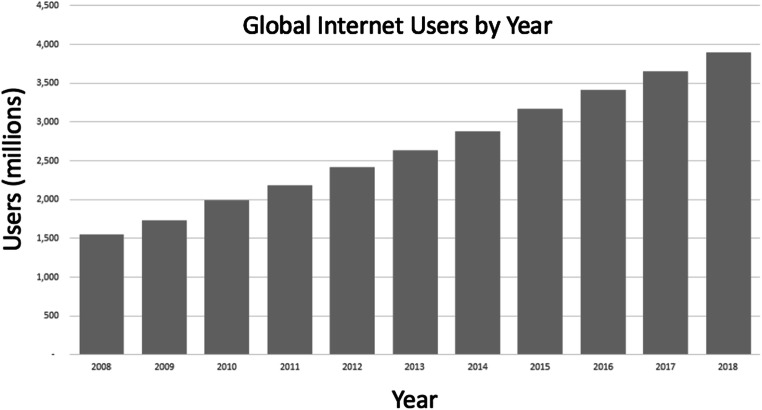
Global internet users over time; data taken from the UN International Telecommunication Union (https://www.itu.int/en/ITU-D/Statistics/Pages/stat/default.aspx)

The access individuals have to the internet via a personal or shared device has also increased over this time, and continues to increase relatively linearly. This is illustrated in Fig. 2, using data provided by the United Nations International Telecommunication Union. This pattern indicates a continuing increase in the potential reach of any web-based research to larger proportions of populations across the globe. This is particularly important considering a historical problem with under-powered research leading to unreliable results, where increased sample sizes provide one way to address this issue (Button et al., 2013).

### The current state

Despite the potential availability of large samples online, there is a hesitancy to adopt certain types of tasks and experiments, particularly those that utilise short stimulus durations (e.g. visual masking experiments) or that need very accurate response time logging (such as an attentional flanker task). The relative noise from online studies can be characterised as coming from two independent sources:

1. Differences in participant behaviour relative to a lab setting
2. Differences in technology, such as software (OS, web browsers, and platforms) and hardware (screens, computers, mobile devices)

The differences in participant behaviour when taking part remotely is difficult to address systematically with software or hardware, and ultimately comes down to the design of the experiment, and utilisation of certain tools. That being said, there *are* ways to reduce this noise—a brief summary of how to improve the quality of data collected online is given by Rodd (2019), and is also discussed in Clifford & Jerit (2014) and more recently in a tutorial by Sauter, Draschkow, & Mack (2020). This paper, however, focuses on issues related to the second point: measurement error introduced by technology. This issue *can* be improved through restriction of hardware and software, and quantifying the introduced imprecisions would help reassure researchers, enabling them to utilise large web-based samples easily in timing-sensitive experiments.

There have been various claims made on the scientific record regarding the display and response timing ability of experimental set-ups using web browsers—for instance, that timing can be good depending on device and set-up (Pronk, Wiers, Molenkamp, & Murre, 2019), and that different techniques for rendering animations lead to reduced timing precision (Garaizar & Reips, 2019). Ultimately, though, the variance in timing reflects the number of different ways to create an online experiment, and the state of the software and hardware landscape at the time of assessment—all of these are changing at a fast rate. We previously undertook a discussion of the changing hardware and software ecosystem in Anwyl-Irvine et al. (2019). To address this variance, it is important to report any timing validation on a range of devices. To the authors’ knowledge, the largest number of devices tested with online software was undertaken by Reimers and Stewart (2015), where 19 Windows machines were assessed, and it is suggested that systems (OS and devices) contribute the greatest variability, with Windows XP displaying less variability than Windows 7. The justification for only testing Windows devices was that 85–90% of their participants used these. However, this has changed since 2015; see the demographics section of this paper for more details.

In a highly commendable concurrent effort, Bridges, Pitiot, MacAskill, and Peirce (2020) compare a wide range of online and offline experimental software across several different operating systems (Windows, Linux, and macOS) and web browsers (Chrome, Edge, Edge-Chromium, Firefox, and Safari). Their data paint an encouraging picture, with reaction time (RT) lags of 8–67 ms, precision of < 1 ms to 8 ms, visual lagging of 0–2 frames, and a variance of under 10 ms for most combinations. Auditory lag is poorer across the board, with average delays ranging in the hundreds of milliseconds. Our study asks similar questions, and uses a similar approach as theirs, with a few crucial differences. Firstly, Bridges et al. (2020) employed a test that is highly suitable for testing lab environments, whereas we aimed to realistically simulate participants' home environments by using an actuator to perform presses on keyboards (Bridges and colleagues employed a high-precision USB button box). Secondly, the authors only assessed one frame duration (200 ms), so they were not sensitive to any interaction between duration and timing errors, whereas we assess 29 different durations. Thirdly, the authors used a lower number of trials for their duration tests than we do (1000 vs 4350), and were therefore less likely to detect irregular delays. Nevertheless, our two concurrent studies have come to similar conclusions, with some differences and limitations to ecological validity in both studies that are further explored in the Discussion. Together, the two studies provide a richer picture of the current state of affairs than each would alone.

A vital issue with research into timing is that it is tempting to interpret results from one (or a set of) studies, and extrapolate this to all ‘online research’. However, most online research is undertaken using different builders, hosting websites, and entire software-as-a-service (SaaS) platforms; very little is made using written-from-scratch JavaScript. These different platforms and websites are separate software, each providing different animation, rendering and response polling code. Just because good timing is possible using one particular JavaScript method in a specific scenario does not mean that it will be great in all online studies. Therefore, in this paper, we compare a variety of online study platforms.

### A realistic approach to chronometry

Researchers must be furnished with the information they need to make sensible decisions about the limitations of browsers, devices, and operating systems. With this information, they can trade off the size of their participant pool with the accuracy and precision of the collected data. If we are to make any timing validation functionally informative to the users, we have to ensure that our methods are representative of the real-world set-ups that our participants will be using. Failure to do so could result in unexpected behaviour, even when running previously well-replicated experiments (Plant, 2016).

When researchers assess the accuracy of software in respect to timing, often the software and hardware set-ups are adjusted significantly in order to record optimum performance in the most ideal environment. These set-ups require the removal of keyboard keys and soldering on of wires (Reimers & Stewart, 2015) or specialised button boxes (Bridges et al., 2020), and include discrete graphics cards (Garaizar, Vadillo, & López-de-Ipiña, 2014; Bridges et al., 2020). This does not represent the average internet user's devices at all. For instance, in the first quarter of 2019, less than 30% of new PCs sold included discrete (i.e. non-integrated) graphics cards (Peddie, 2019), likely representing an even smaller number of online participants. Recently, Pronk et al. (2019) utilised a robotic actuator to press keyboard keys and touchscreens, a more representative assessment of RT recording. Testing on ideal-case set-ups, whilst vital for realising the frontier of what is possible with online software, is likely to poorly reflect the situation researchers face when collecting data online. Consequently, we have made an attempt to use more realistic set-ups in our study, such as an actuator on consumer keyboards in our research.

The first and second parts of this paper test the visual display and response logging performance of different software on different common browsers and devices, in order to give an indication of each set-up’s limits. The final part of the paper then provides an overview of the device demographics of online participants, with a snapshot sample of over 200,000 Gorilla participants taken in 2019. Pronk et al. (2019) use global web user data to select the browsers they use, but this may be different from the sub-population of those who engage in online research. Our approach is therefore well-suited to estimate the distribution and variability of devices and browsers within the online participant population.

For the testing sections, we selected a realistic variety of devices. Windows and macOS operating systems cover the majority of the population for online testing (73% of our user sample). The devices we use are split between a desktop PC with an external monitor, a desktop Mac with an integrated monitor, a high-spec Windows Ultrabook, and a lightweight Mac laptop. Further to this, the devices are assessed as they are, with no steps taken to restrict the browsers or operating systems, increasing the likelihood that they reflect users’ actual set-ups.

In order to provide researchers a barometer of how their participants’ devices will perform, we have endeavoured to cover as many commonly used tools, operating systems, and devices as possible (given the number of trials needed for each test). We have assessed these using an external chronometry device that can independently capture the accuracy and precision of systems.

We also distinguish between the *average* accuracy of the timing of set-ups (e.g. on average, how close to the actual reaction time is a given set-up’s record) and the *variability* of this accuracy (i.e. will the reaction time error vary a great deal within one experiment). Variability in presentation and reaction times increases the noise in the experiment. For example, a delayed—but consistent—reaction time record permits comparisons between trials and conditions, whereas variability in this can potentially obscure small differences between conditions. These concepts are referred to respectively as *accuracy* and *precision.*

In all data reporting, we have intentionally avoided the use of inferential statistics, and chosen to show descriptive statistics, an approach previous studies have taken (Neath et al., 2011; Reimers & Stewart, 2015, 2016). We made this choice for two reasons. Firstly, the distributions of the data traces produced are highly irregular, and deviations are either very small and frequent or very large and infrequent, making formal comparison very difficult. Secondly, there is no ideal way to define a unit of observation. If we consider each sample within a condition, the large number of samples is likely to make any minor difference statistically significant, even if it is not practically meaningful. Alternatively, if we consider each device-browser-platform combination, comparisons would be severely under-powered. We thus report descriptive statistics, as well as the entire distribution of samples within each cell.

We undertake three analyses in this paper to answer the questions of accuracy and precision in realistic set-ups. The first deals with the timing of visual stimuli presented on a screen, where the delay we report is the difference between the expected duration on the screen versus the actual duration. The second characterises the accuracy of each set-up in recording keyboard presses in response to a displayed item on-screen, where the delay is the difference between the recorded press onset and the actual onset. The third characterises the participants themselves: what devices they use, where they are based, and what recruitment services are used—this provides context to our results.

## Visual duration accuracy

This experiment looks at how robust different web-based tools are when it comes to both response recording and display accuracy. We compare our platform, *Gorilla v.20190828,* with three other web-based tools: *jsPsych v6.0.5*, *psychoJS/PsychoPy3* v3.1.5 (produced from their builder and hosted on *Pavlovia.org*), and *Lab.js v19.1.0* (using their builder).

These implementations are tested in a variety of configurations to represent some of the most common participant scenarios. Five browsers are used: *Chrome, Firefox, Edge, Safari, IE*; and two operating systems are used, *Windows 10* and *macOS Mojave.*

### Methods

#### Display duration

The visual duration delay (VDD) experiment assessed the accuracy of the platform’s visual display timing on the test rigs. A series of white squares were presented for a variable duration on a black background, with a 500-ms/30-frame inter-stimulus interval. Stimuli were presented for a duration of 1–29 frames (1/60th of a second to one-half of a second) to create a profiling trace for each system. Each duration was repeated 150 times, for a total of 4350 presentations per hardware and software combination. The order of these durations was randomised. The white and black squares were PNG images, and were identical for each platform.

We constructed each task according to each platform’s documentation. The details are described for each platform below:

##### Gorilla v.20190828

A task was created with two screens, both containing an ‘image zone’ and a ‘timing zone’. The first zone was configured to show the black PNG image, and the second a white PNG image; these were uploaded as stimuli to Gorilla v.20190828. The timing zone was set to read information from a configuration spreadsheet containing the timings described above; the duration was variable for the white PNG and set to 500 ms for the black PNG. Fullscreen was enabled by requesting this in the ‘onScreenStart’ function in the Task Builder. The task was run in the browser using the ‘Preview’ button.

##### jsPsych v6.0.5

jsPsych v6.0.5 had a GUI builder at the time of running the experiment; however, we did not use it, as this was still in beta, and we wanted to assess the most common implementation at the time. The black and white PNG images were presented using the ‘image-keyboard-response’ plugin, with black and white trials alternating, and both the ‘stimulus_duration’ and ‘trial_duration’ were set from a series of ‘timeline_variables’ to hold the durations described above. These were randomised by setting the ‘randomize_order’ value to ‘true’. The task was run locally by opening up an HTML file containing the JavaScript and importing the toolbox using script tags. As jsPsych was set to pre-load assets and scripts, running the task locally would not result in differences compared to running on a remote server (like the Gorilla and PsychoJS examples). The fullscreen plugin was used to request the fullscreen window.

##### psychoJS/PsychoPy3 v3.1.5

We used the PsychoPy3 v3.1.5 Builder GUI to construct this task. A trial was created containing two image stimuli, one the black PNG image and the other the white PNG image. The black stimulus had a start time of 0.0 s and a stop time of 0.5 s; the white stimulus had a start time of 0.5 s and a stop duration of a variable value described above (referred to in the builder using the ‘$’ syntax). These trials were presented using a loop within a builder routine, the CSV containing durations for the white trial was specified in the ‘Conditions’ field of the loop properties, and the ‘loopType’ was set to ‘random’. The task requested fullscreen; this was done using the experiment settings in the builder—in the JavaScript code, the openWindow ‘fullscr’ attribute is set to ‘true’. The task was then exported to PsychoJS v3.1.5 and uploaded to Pavlovia.org using the GUI, where the task was run from a browser.

##### Lab.js v19.1.0

The task was created in Lab.js v19.1.0’s in-browser GUI builder tool. A frame was used containing an HTML canvas. A ‘Loop’ was created with the spreadsheet variables uploaded, containing the required durations of the white squares. Within the loop a ‘Sequence’ was created, which contained two components, one with the black PNG and one with the white PNG, both uploaded as ‘media’ in the Content tab. The timeout field in the Behaviour tab for the black PNG was set as 500 ms, and the field value was taken from the loop for the white PNG. Fullscreen was requested using the fullscreen pre-made class on the canvas.

The task was then exported for local use, using the offline data collection option in the ‘save’ menu. As Lab.js pre-loads assets and scripts, running the task locally would not result in differences compared to running on a remote server (like the Gorilla and PsychoJS examples).

The duration of each white square was recorded using a photodiode/opto-detector connected to a Black Box Toolkit version 2 (BBTKv2) (Plant, 2014). This photo-diode was attached to the centre of each screen with an elastic strap, ensuring it was attached firmly and flatly to the screen. In line with the BBTKv2 user manual, an amplitude threshold was used that was relative to each screen. This was titrated beforehand with a continuously flashing square, and the highest threshold that permitted detection of the flashing white square was chosen.

#### Browsers

Browser versions were verified from the browsers themselves on each machine rather than via version tracking tools within testing platforms, as these were sometimes inaccurate, or used different versioning conventions (e.g. Edge 44 on Windows 10 desktop PC was recorded as Edge 18.17763 by Gorilla—the first being the version of the browser and the second being the HTML engine version). The browser versions used were Chrome 76 (Windows), Chrome 75 (macOS), Firefox 68 (Windows), Firefox 69 (macOS), Safari 12 (macOS), and Edge 44 (Windows).

At the time of testing, PsychoJS v3.1.5 would not run on Edge on our set-ups; this compatibility issue has been fixed, but we were unable to test this set-up, as this was a recent development and would require re-testing all platforms to be equitable, which is not feasible due to the resources needed.

#### Devices

The two devices were (1) a Windows desktop running Windows 10 Pro, with an Intel Core i5-2500 3.3 GHz CPU, 8 Gb of RAM, and a 60 Hz ASUS VS247 23.6” monitor with 1920 × 1090 resolution; and (2) a 2017 Apple iMac running macOS 10.14.1 with an Intel Core i5-7400 3.0 GHz CPU, a built-in 21.5” monitor with a 4096 × 2304 resolution. The devices used were not adjusted or restricted in any way. This meant that background processes such as virus scans and file-sharing services could spike in activity during the study, just as they could on a participant’s computer.

#### Platforms

All data were collected between June and September 2019. The Gorilla task was run on builds 20190625, 20190730, and 20190828, and the PsychoJS task was made with PsychoPy3 v3.1.5 and hosted on Pavlovia.org—this was up to date at the time of testing, although a newer version has since become available, and is reported to have better timing (Bridges et al., 2020). The jsPsych task was made using v6.0.5. The Lab.js task was built using the GUI, and was made with version 19.1.0.

#### Data processing

The metric of interest is the accuracy and precision of displaying the white square for the requested duration. This can be expressed as a delay score where the expected duration of the square and the actual recorded time from the photodiode are compared in milliseconds. Outliers (defined as more than four standard deviations from the mean) were included in the plots, and their range is reported, as we believe these very rare trials may still be of interest. Occasionally, on under-presentation, durations of a single frame would not be rendered, leading to a continuous black square; these had to be manually accounted for in the analysis by replacing the missing opto-detector value with a ‘0’, and were identified when the opto-detector recordings became offset by 1.

### Results

**Fig. 3:**
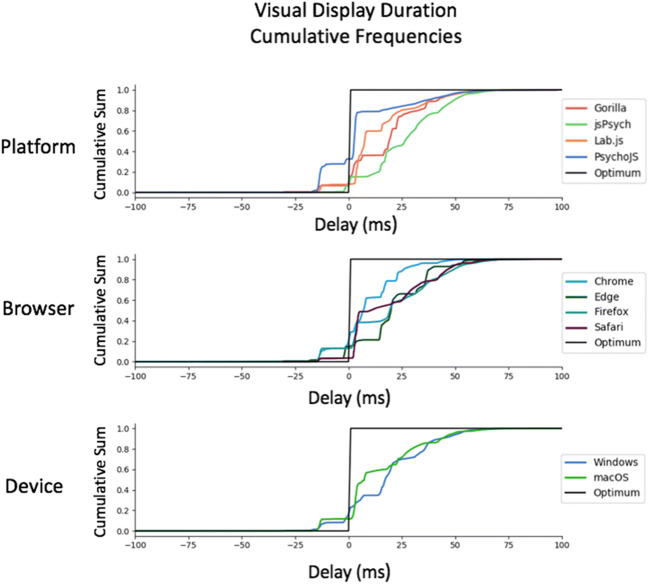
Cumulative frequency plots for delays in visual duration, separated by testing platform (top panel), browser (middle panel), and operating system (bottom panel). (Gorilla versions 20190625, 20190730, and 20190828; Lab.js version 19.1.0; PsychoJS/PsychoPy version 3.1.5; jsPsych version 6.0.5)

**Fig. 4:**
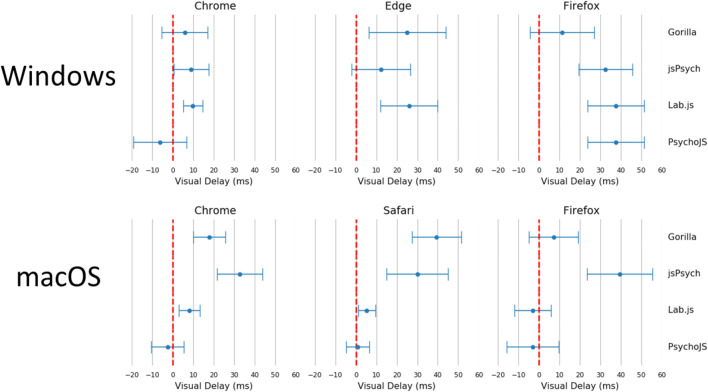
Average visual delay across all frame lengths, broken down by browser, platform, and operating system. Each point represents the average, with bars representing the standard error across all frames. (Gorilla versions 20190625, 20190730, and 20190828; Lab.js version 19.1.0; PsychoJS/PsychoPy version 3.1.5; jsPsych version 6.0.5)

Summary statistics for this test are shown in Table 1**.** The cumulative distributions of these summary statistics are also illustrated in Fig. 3. Figure 4 shows a summary of these delays on the level of individual testing sessions, with the standard error and mean for each combination. We have not converted all timings to frames, and have summarised the data in milliseconds for transparency, as the iMac screen appeared to not always stick to 60 Hz. All platforms exhibited a positive delay (on average, they overrepresented the duration of items), except for PsychoJS v3.1.5, which both overestimated and underestimated. In terms of timing, *Chrome* and *Windows* appear to show the smallest delay. In terms of variance, the smallest standard deviation was with *Lab.js v19.1.0*, which had a maximum delay of 16.49 ms (one frame at 60 Hz) and an average of 9.8 ms. The other platforms appear to exhibit almost equivalent delay. Browsers and platforms showed no superiority in terms of variance.

**Table 1.** Summary of Visual Duration Delay results in milliseconds. Visual Duration Delay is calculated as the difference in milliseconds between the requested duration of a white square and the duration that is recorded by a photodiode sensor. It is broken down by Platform (Gorilla versions 20190625, 20190730, and 20190828; Lab.js version 19.1.0; PsychoJS/PsychoPy version 3.1.5; jsPsych version 6.0.5), Browser, and Device

**Visual Duration Delay**					
**Platform**	***Mean***	***Standard Deviation***	**Percentiles**		
			***25%***	***50%***	***75%***
*Gorilla*	13.44	15.41	2.25	17.50	22.50
*Lab.js*	9.79	4.69	6.17	6.90	15.70
*PsychoJS*	-6.24	12.99	-13.00	-10.75	-1.00
*jsPsych*	26.02	17.40	15.25	26.50	37.00
**Browser**	***Mean***	***Standard Deviation***	***Percentiles***		
			***25%***	***50%***	***75%***
*Chrome*	11.50	15.40	0.50	7.22	22.75
*Edge*	18.72	17.88	3.50	18.75	32.25
*Firefox*	22.58	19.80	3.50	19.75	36.25
*Safari*	30.02	15.07	17.50	29.25	42.50
**Device**	***Mean***	***Standard Deviation***	***Percentiles***		
			***25%***	***50%***	***75%***
*Windows*	12.43	17.11	0.50	15.25	19.75
*macOS*	25.45	17.17	14.25	23.50	34.50

**Fig. 5:**
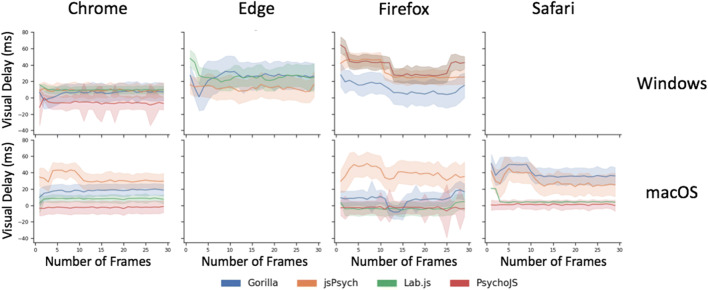
Visual delay traces broken down by web browser, operating system, and platform. Visual delay is the delta between requested and recording duration in milliseconds, shown across 30 frames. The shaded errors represent standard error. Safari on Windows, and Edge on macOS, are not supported (so missing). (Gorilla versions 20190625, 20190730, and 20190828; Lab.js version 19.1.0; PsychoJS/PsychoPy version 3.1.5; jsPsych version 6.0.5)

**Fig. 6:**
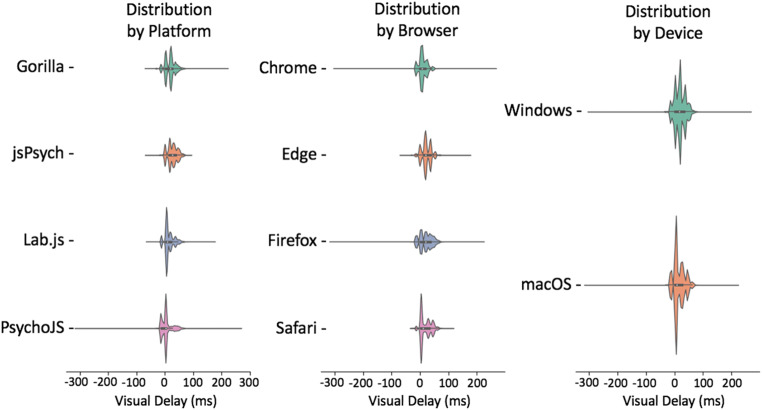
Visual delay violin plots of data broken down by platform, browser, and device. The shaded error represents the distribution density, the lines represent the span of times, and the white dot represents the mean. (Gorilla versions 20190625, 20190730, and 20190828; Lab.js version 19.1.0; PsychoJS/PsychoPy version 3.1.5; jsPsych version 6.0.5)

A more fine-grained overview of the results for VDD can be seen in Fig. 5. The overall story is complex, with traces varying in shape, but some themes are apparent. In macOS, across different devices and platforms, jsPsych v6.0.5 consistently showed a slight delay for requested durations between 3 and 20 frames. Firefox showed the largest amount of variance out of all the browsers, both between different frame lengths **(**Fig. 5**)** and between different platforms (Fig. 4), leading to a more drawn out distribution in Fig. 6. The best all-round browser was Chrome— it showed the least variance across devices and platforms, although it was more spread out between platforms on macOS (Fig. 4).

The traces in Fig. 5 also tell us that delays persist in longer durations as well as shorter durations: in most platforms, the error at one frame (16.66 ms) was the same as the error at 30 frames (500 ms). This is positive for users who wish to conduct research with different durations for different images, and means that variability will be broadly equivalent between times. The exceptions to this are jsPsych v6.0.5, Firefox, and Edge, which should probably be avoided in this scenario.

Outliers are very rare, with 22 trials out of 103,500. They range from 95.75 to 265 ms. They are fairly equally distributed among some platforms (10 Gorilla, 9 PsychoJS v3.1.5, 4 Lab.js v19.1.0, 0 jsPsych v6.0.5), but it is difficult to draw inferences from so few instances. These are likely due to display or external chronometery anomalies—it is difficult to tell with such low rates of replication.

We note that the above descriptions relate to the data collected from tested devices, and would not necessarily generalise to the population of participants’ home devices.

## Reaction time accuracy

This experiment assessed the accuracy of an entire system to record responses on a keyboard. The BBTK robotic actuator was programmed to press a space key in reaction to a white square at a pre-specified reaction time. This actuator uses a motor to propel a metal ‘finger’ with a foam tip onto the keyboard of the device. Once calibrated, it can deliver reaction times with sub-millisecond accuracy. We opted for using an actuator instead of deconstructing a keyboard to attach wires to the underlying board, for two reasons: it enables us to easily test touchscreen devices in the future, and it more closely resembles what participants of online experiments do, without optimising for an unrealistic set-up.

### Methods

#### RT task

As in the VDD experiment, an opto-detector was connected to each system on an elastic band, and connected to the BBTK. This detector acted as a trigger for the actuator to make a response, programmed with a fixed reaction time of either 100, 200, 300, or 500 ms representing a reasonable range of fast responses by human participants. As in the VDD experiment, the opto-detector threshold was adjusted to suit each screen and set-up. The actuator was calibrated before each testing run, using a TTL trigger button provided as part of the BBTK kit. Ten presses on this button give an initiation latency for the actuator, and this latency is accounted for when programming the key presses.

Some software tools force a fullscreen by default on certain operating systems (e.g. Lab.js v19.1.0 on Safari on macOS), which caused a white flash between setting the photodiode as a trigger and the experiment starting. This potential measurement problem was addressed by adding an extra single 10 ms press of the actuator (not long enough to touch a key) before the main task, so that the initial flash did not impact the rest of the task. Very rarely (occurring only twice during all of the tests) the actuator would fail to be triggered by the opto-detector, or the keypress would not be registered. These trials were excluded from the analysis.

We constructed each task according to each platform’s documentation, using a GUI whenever possible. Most details are identical to the first experiment above; below we describe the key changes made for the RT experiment:

##### Gorilla

The screens were identical to Experiment 1, but with a ‘Response Keyboard’ zone replacing the ‘Timelimit’ zone—which ended the trial when the key was pressed.

##### jsPsych v6.0.5

The task was identical to Experiment 1, except that there was no time limit on the white PNG trial, and the ‘response_ends_trial’ was set to ‘true’.

##### psychoJS/PsychoPy3 v3.1.5

The task was identical to Experiment 1, except a key response component was added, with the default options set, and the duration of the white PNG not set.

##### Lab.js v19.1.0

The task was identical to Experiment 1, except the timeout was set to ‘never’ for the white PNG image and a keydown response was added.

#### Browsers

As in the VDD test, we did not want to configure the browsers in any way beyond a standard user set-up, so there was very minor variance in versions. The browser versions used were as follows: macOS desktop: Chrome 76, Firefox 69, Safari 12; macOS laptop: Chrome 75, Firefox 69, Safari 11; Windows desktop: Chrome 76, Firefox 68, Edge 44; Windows laptop: Chrome 75, Firefox 67, Edge 44.

At the time of testing, PsychoJS v3.1.5 would not run on Edge on our set-ups; this compatibility issue has been fixed, and we hope to include these data in a future version of this paper.

#### Devices

The two desktops were (1) a Windows desktop running Windows 10 Pro, with an Intel Core i5-2500 3.3 GHz CPU, 8 Gb of RAM, and a 60 Hz ASUS VS247 23.6” monitor with 1920 × 1090 resolution; and (2) a 2017 Apple iMac running macOS 10.14.1 with an Intel Core i5-7400 3.0 GHz CPU, a built-in 21.5” monitor with 4096 × 2304 resolution.

Because laptops have different configurations of keyboards compared to desktops (i.e. they are connected internally rather than through USB), we employed two in this experiment. These were (1) a Windows Surface Laptop 2, with an Intel core i7 CPU, 16 Gb RAM, and an integrated touchscreen 13.5” 60 Hz display with a 2256 × 1504 resolution; and 2) a MacBook Air early 2016, running macOS 10.14.1 with an Intel Core m5 1.2 Ghz CPU, 8 Gb RAM, with a 12” 60 Hz Retina display with 2304 × 1440 resolution.

The devices used were not adjusted or restricted in any way. This meant that background processes such as virus scans and file-sharing services could experience a spike in activity during the study, just as they could on a participant’s computer.

#### Platforms

The same versions of experiment software were used as in the VDD.

#### Data processing

The delay scores were calculated as the difference between the known actuator reaction time and the recorded time on the software. No outliers (more than four standard deviations from the mean) were detected.

### Results

**Fig. 7:**
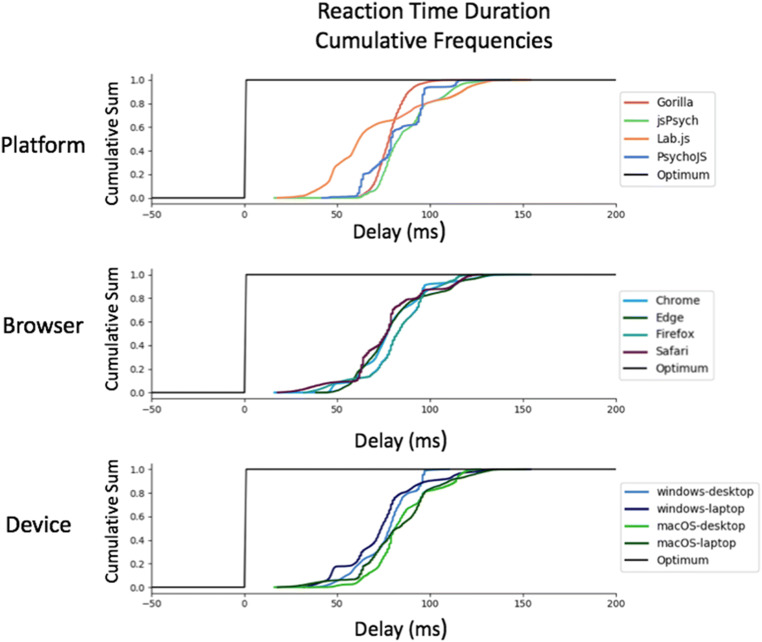
Cumulative frequency plots for delays in visual duration, separated by testing platform (top panel), browser (middle panel), and operating system (bottom panel). (Gorilla versions 20190625, 20190730, and 20190828; Lab.js version 19.1.0; PsychoJS/PsychoPy version 3.1.5; jsPsych version 6.0.5)

**Fig. 8:**
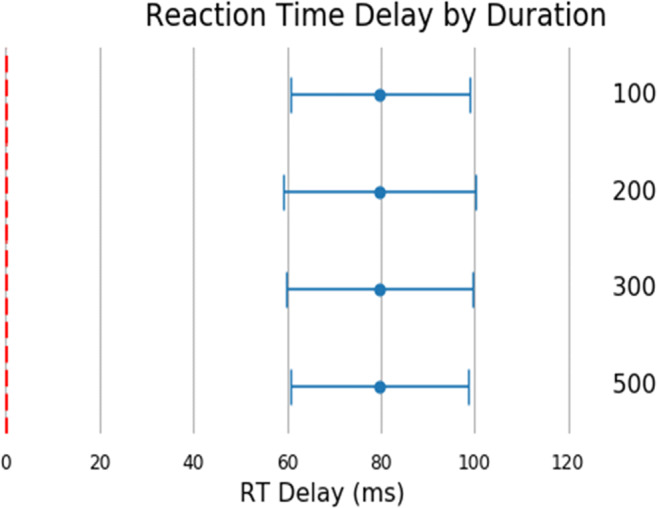
Reaction time delay by requested duration. Points represent the mean, and error bars represent the standard deviation

**Fig. 9:**
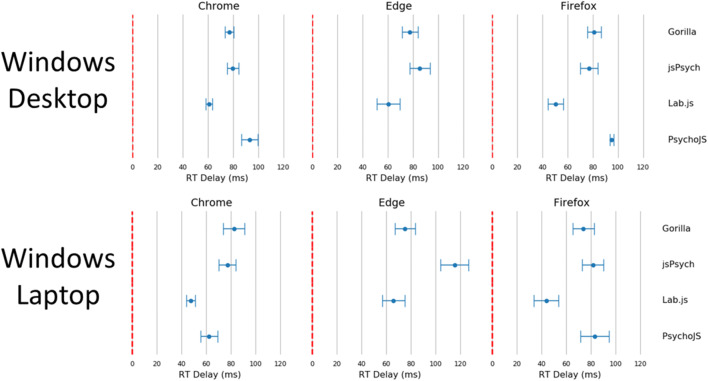
Reaction time delay for Windows 10 devices broken down by browser, device and platform. Points represent the mean, and bars represent the standard deviation. (Gorilla versions 20190625, 20190730, and 20190828; Lab.js version 19.1.0; PsychoJS/PsychoPy version 3.1.5; jsPsych version 6.0.5)

**Fig. 10:**
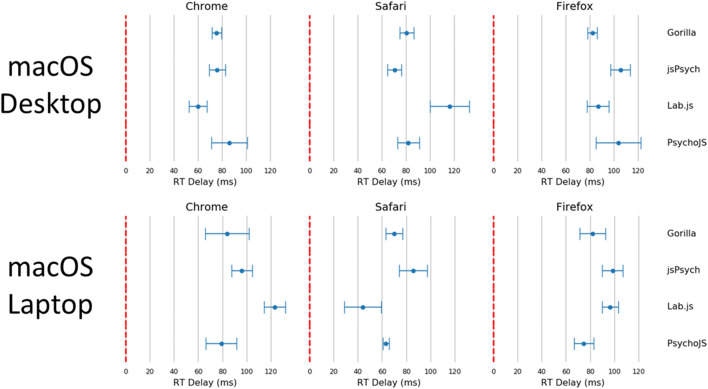
Reaction time delay for macOS devices broken down by browser, device, and platform. Points represent the mean, and bars represent the standard deviation (bottom panel). (Gorilla versions 20190625, 20190730, and 20190828; Lab.js version 19.1.0; PsychoJS/PsychoPy version 3.1.5; jsPsych version 6.0.5)

**Fig 11:**
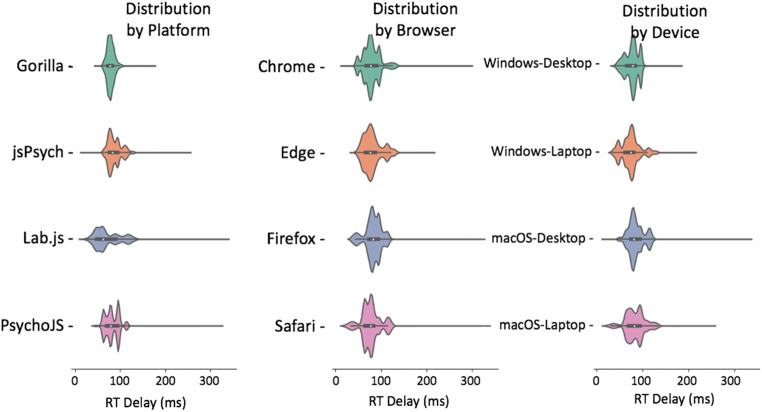
Reaction time violin plots organized by platform, browser, and device. Lines represent the maxima and minima, whereas the shaded error represents a distribution plot (bottom panel). (Gorilla versions 20190625, 20190730, and 20190828; Lab.js version 19.1.0; PsychoJS/PsychoPy version 3.1.5; jsPsych version 6.0.5)

The figures in the above section where replicated for reaction time (Figs. 7, 8, 9, 10, and 11). Reaction time delay (the difference between performed reaction time by the actuator and that recorded on the experiment platform) was broken down by the requested reaction time (100, 200, 300, and 500 ms). This allowed us to investigate whether any particular duration led to more error in systems in general. This was not the case overall (Fig. 8). There were also a few differences between desktop and laptop computers, particularly on Windows. More importantly, experiment platforms did not all behave in similar ways.

Gorilla was relatively consistent overall, with around 80 ms of delay for all operating systems and device types. It also had good precision, with the lowest overall standard deviation out of all the platforms (8.25 ms in Table 2). As discussed above, high precision in measuring reaction times permits a higher sensitivity for small differences between conditions. The platform also showed slightly higher standard deviations for laptops compared to desktop keyboards. However, this was in line with the average results broken down by device type in Table 2**.**

**Table 2:** Summary of reaction time (RT) delay results. RT delay is calculated as the difference between known and recorded RT. It is broken down by platform, browser, and device. All results are reported after outliers have been excluded. (Gorilla versions 20190625, 20190730, and 20190828; Lab.js version 19.1.0; PsychoJS/PsychoPy version 3.1.5; jsPsych version 6.0.5)

**Reaction Time Delay (ms)**					
**Platform**	***Mean***	***Standard Deviation***	***Percentiles***		
			***25%***	***50%***	***75%***
*Gorilla*	78.53	8.25	73.00	78.00	83.15
*Lab.js*	71.33	28.16	48.53	61.95	90.65
*PsychoJS*	82.28	16.36	70.00	79.00	95.00
*jsPsych*	87.40	15.27	76.00	83.14	95.14
**Browser**	***Mean***	***Standard Deviation***	***Percentiles***		
			***25%***	***50%***	***75%***
*Chrome*	78.81	18.51	67.73	77.00	90.25
*Edge*	80.10	19.81	66.09	76.63	87.90
*Firefox*	82.30	18.62	74.00	82.46	94.00
*Safari*	76.50	21.86	64.00	77.00	84.00
**Device**	***Mean***	***Standard Deviation***	***Percentiles***		
			***25%***	***50%***	***75%***
macOS-Desktop	85.35	18.31	75.00	81.00	95.00
macOS-Laptop	83.13	21.38	69.00	82.09	95.00
Windows-Desktop	76.24	14.47	65.73	78.08	84.96
Windows-Laptop	73.65	20.32	62.00	73.90	81.00

jsPsych v6.0.5 was consistent (around 70 ms) on desktop devices running Chrome or Safari, but less so for Firefox on macOS (desktop: around 110 ms, laptop: around 100 ms) and Windows (desktop and laptop: around 80 ms), and for Edge (desktop: around 85 ms, laptop: around 120 ms).

Lab.js v19.1.0 showed a rather distributed pattern across all combinations of devices and browsers.

PsychoJS v3.1.5 was relatively consistent (around 80 ms) on a macOS, with the exceptions of Firefox on a desktop (around 100 ms) and Safari on a laptop (around 65 ms). It was also consistent on Windows desktop devices (around 95 ms) for Chrome and Firefox, but less so on the laptop (around 60 ms on Chrome, and 80 on Firefox). PsychoJS v3.1.5 also shows clustering around 16 ms increments, likely due to RT logging within the animation loop at the time of testing. We understand that updates made in late 2019 have changed this.

We note that the above descriptions relate to the data collected from tested devices, and would not necessarily generalise to the population of participants’ home devices.

## Participant analysis

### Methods

The data were gathered using Gorilla’s user analytics—those who accessed the website to take part in experiments. This totalled 202,600 accesses. The logs are produced by combining IP address information (e.g. server location, operating system), referring website (e.g. MTurk, Prolific, etc.), and information provided by the client’s web browser (e.g. browser used, screen dimensions). The resulting information is displayed using descriptive statistics and graphs, as an overview of the web demographics of a representative sample of Gorilla’s participant population for that year.

### Results

#### Operating systems and browsers

**Fig. 12:**
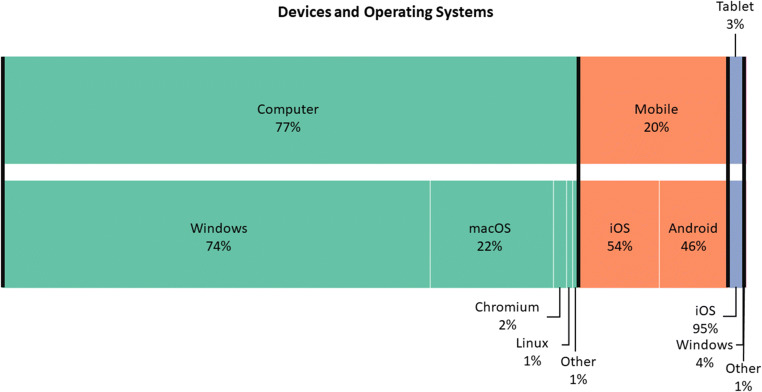
Operating systems and devices, nested and stacked bar chart. Based on a sample of 202,600 participants. Percentages are rounded to the nearest integer

The first thing to consider is the types of devices users are utilising to access the internet. We found that 77% of these devices were desktop or laptop computers, whereas only 20% were mobile devices, and just over 2% were tablets. A more detailed breakdown of the operating systems in use can be seen in Fig. 12. The most common operating system was Windows, followed by macOS. For mobile devices, users were roughly evenly split between iOS and Android, and the overwhelming majority of tablets were iPads running iOS.

**Fig. 13:**
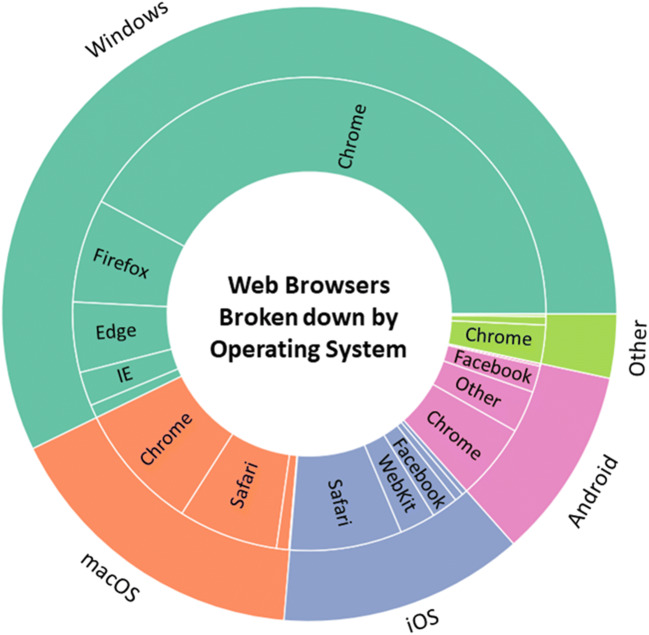
Nested pie chart representing the breakdown of browsers within each operating system. For readability, wedges less than 3% are not labelled, but all are in the ‘other’ category

Table 3 and Fig. 13 show the breakdown of participants’ browsers by operating system. The most common browser was Chrome (59%), but this dominance varied depending on device (it was less popular on mobile operating systems). Overall, the average percentages for Chrome, Firefox, and Safari were in line with what we would expect from the global market share of 64.3%, 16.7%, and 4.5%, respectively (Browser Market Share Worldwide, 2019). Where our sample differs is in the use of the Facebook browser (3.6%), which is not listed in the aforementioned market share statistics. It is likely to reflect researchers sharing studies in the mobile application Facebook Messenger, which opens links with its built-in browser by default.

**Table 3:** Browser percentage broken down by operating system. The averages are shown in bold for each browser. Each is taken from the total data, so are not an average of the operating systems—which have unequal numbers of users

Operating System	*Chrome*	*Firefox*	*Safari*	*Edge*	*Internet Explorer*	*Facebook*	*Webkit*	*Other*
*Windows*	73.60%	12.40%	-	8.30%	4.10%	-	-	1.60%
*macOS*	53.10%	5.10%	41.40%	-	-	-	-	0.40%
*iOS*	-	-	61.20%	-	-	14.50%	19.60%	4.70%
*Android*	51.80%	1.70%	-	-	-	18.00%	-	28.50%
*Other*	77.80%	17.10%	-	0.40%	-	-	-	4.70%
**Average**	**59.00%**	**8.60%**	**14.50%**	**4.80%**	**2.30%**	**3.60%**	**2.40%**	**4.60%**

#### Screen size and window

The screen size of the devices limits the objective size of the items presented on screen. Stimuli size, whilst less important for some phenomena such as visual object priming (Biederman & Cooper, 1992) or perceptual learning (Furmanski & Engel, 2000), is important for others, in particular in some visual perceptual research—for example visual crowding (Tripathy & Cavanagh, 2002)—where it can impact the detectability of targets. We therefore looked at the variation and distribution of the participants’ screen sizes.

It makes sense to analyse computers, mobile devices, and tablets separately, as experimenters interested in size are likely to restrict themselves to one of these categories. The two most common screen sizes for computers were 1366 × 768 pixels (23.2%) and 1920 × 1080 pixels (21.5%); for mobile devices these are 375 × 667 pixels (27.8%) and 360 × 640 pixels (18.5%)—both in portrait mode; and finally tablets with 768 × 1024 (73.7%)—the logical resolution of all iPad minis and iPad airs.

**Fig. 14:**
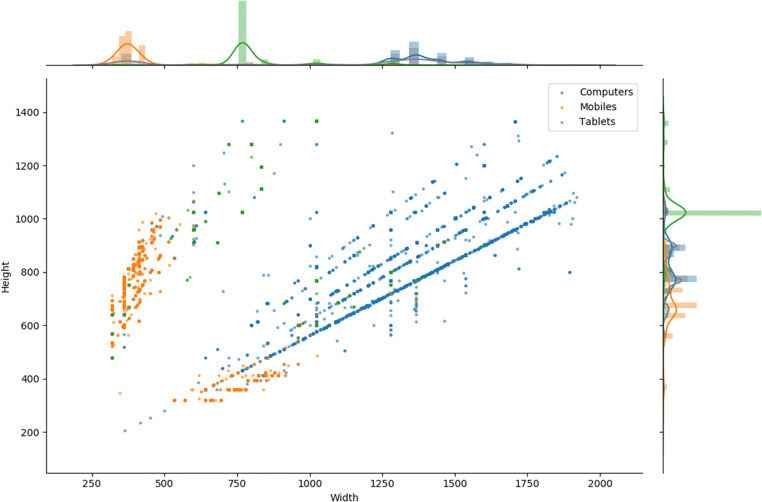
Scatter graph of screen width and height, with histograms and kernel density estimation plots for each dimension. The diagonal lines represent the different aspect ratios

Looking at the most frequent resolution combinations tells only part of the story; it becomes more interesting when we translate size into a continuous variable and look at the distribution of screen dimensions. This is illustrated in the scatter graph in Fig. 14. The mean width of computer screens was 1387.6 pixels (SD = 161.9), and the height was 832.3 pixels (SD = 99.5); mobile screens had a mean width of 393.2 pixels (SD = 92.4) and a height of 684 pixels (SD=109.5); tablets had a mean width of 811.7 pixels (SD = 141.1) and the height was 1025 pixels (SD = 128). The variance in tablets and mobile devices is likely overestimated, as the screen sizes are split between landscape and portrait users. This landscape/portrait split is illustrated in Fig. 14, where tablets and mobile points appear to mirror each other in clusters.

Figure 14 also nicely shows the differing aspect ratios present in computers—with strong diagonal lines along those ratios (as the screens scale up with those rules). The most common aspect ratio was 16:9 / 1.77—41% of computers show this, and it scales up along the thickest blue line. There are also less clear aspect ratio lines for mobile devices.

**Fig. 15:**
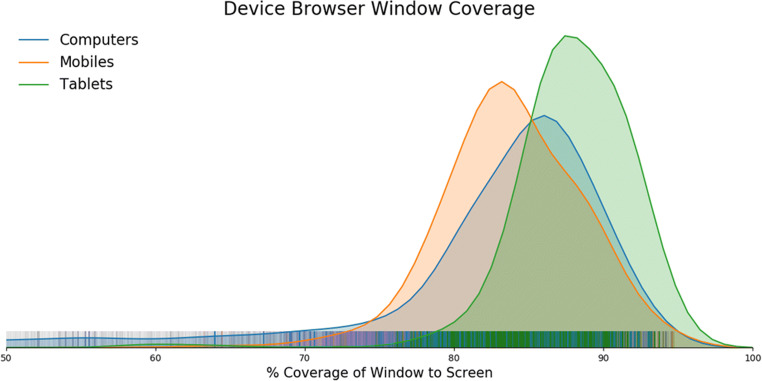
Kernel density estimation of browser window coverage relative to screen size, with individual points as a carpet plot

Screen size does not account for the entire presentation of stimuli on-screen. The browser window will always take up less than 100% unless the user is in fullscreen mode. We quantified this in our sample by calculating the percentage coverage the browser window had on each screen. This can be seen illustrated in Fig. 15. Computers have a longer tail of coverage, as users are able to scale the window with their mouse—something not as easy in tablets (highest coverage) and mobile devices (slightly less).

#### Geography

**Fig. 16:**
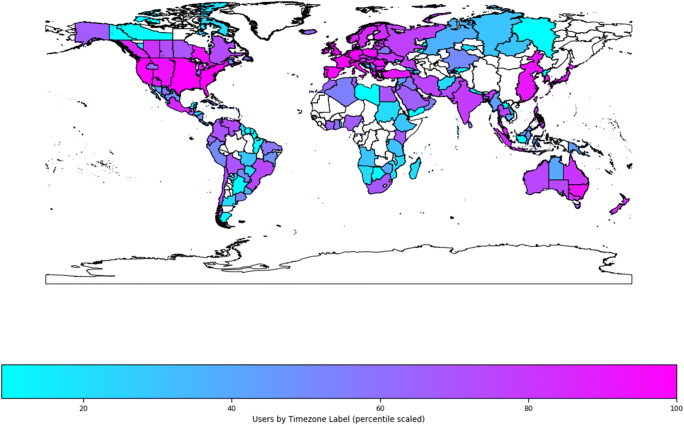
Time zones of participants. The data are scaled into percentile rank scores within the whole sample, for interpretability of geographical spread (but not relative contribution)

We estimated geographical location from participants’ time zone data. These were recorded in a standard format, and obtained using *moment.js (*https://momentjs.com/timezone/docs/). The labels produced refer to time zone localities, according to the TZ Database (Lear & Eggert, 2012). Seventy percent (over 131,000) of the participants were based in Europe (mostly in the UK: 53%, the Netherlands: 3%, Germany: 2%, and France: 1%), and 23% (over 44,000) were based in the American continent (mostly in TZ codes New_York: 10%, Chicago: 5%, and Los_Angeles: 3%). The distribution (Fig. 16) is heavily biased towards westernised developed economies, which is not reflective of the broader internet-using population, the majority of which is based in Asia (57%) (International Telecommunication Union, 2019).

**Fig. 17:**
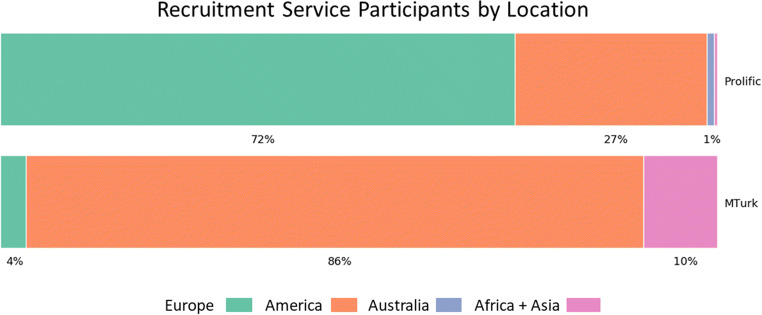
Continents of participants from each recruitment platform. Africa and Asia are combined as they represent a relatively small number of participants

We were able to look at the geographical distribution of participants tested using different recruitment services; a breakdown between MTurk and Prolific is shown in Fig. 17. Prolific (previously Prolific Academic) is an online study panel which specifically targets research participants rather than professional survey responders and human training for machine learning (Palan & Schitter, 2018). As such, its participant demographics in our sample (based on machine timestamp) are more heavily skewed towards Europe and America—but with Europe being the dominant area—whilst MTurk seems to show a large number of users from America, but also a relatively increased number from Africa and Asia. This difference could represent a difference in panel demographics, or it could reflect researchers’ criteria for recruitment within these websites.

#### Limitations

As the data presented here are restricted to researchers who used the Gorilla platform, it reflects the recruitment practices and potential restrictions of their research projects. This caveat is important when we consider the browser and mobile device breakdown, as it is possible that researchers used Gorilla’s GUI tools to restrict participants in some way. Unfortunately, we do not log these aspects of researchers’ usage. Because browser vendors do not consistently report OS for security reasons, Gorilla is unable to reliably restrict by operating system, so this aspect of our data is likely to be representative. Our sample size is large here—over 200,000 participant devices—so it is conceivable we would have a reasonable degree of overlap with the general online participant pool. Specifically, 43% of the sample were recruited using a simple link, and these are likely to be representative of online participants generally; 33.2% were recruited from Prolific and are likely to be representative of Prolific users; 23.8% were recruited using MTurk and are likely to be representative of MTurk workers.

### Discussion

We undertook timing validation of presentation and response times on common browsers, platforms, and devices. Encouragingly, all platforms are reasonably accurate and reliable for studies not needing < 100 ms reaction time accuracy or < 2 frames presentation accuracy. However, we reveal complex patterns of variation between all set-up variables, and in general show that experiment platforms do not behave consistently between browsers and operating systems. We also conducted an analysis of 200,000 online research participants, and found that some demographic factors do not overlap with the general online user population, and that choice of recruitment method impacts one’s population. The device, browser, and geographical distributions of online participants reported here could help researchers make sampling decisions.

We found that the choice of platform contributes greater variance than the device—contrary to earlier findings that systems introduced more variance than browsers (Reimers & Stewart, 2015). This is likely because browser technology has changed quickly in the past few years—as discussed in the introduction—and how platforms manage and render stimuli has also changed. Due to the huge number of trials we had to conduct, it was not feasible to undertake testing on more than the four devices assessed here, but it is perhaps worth replicating this analysis on a wider range of devices, such as touchscreen Android and iOS (despite these devices accounting for a smaller proportion of users in our sample). It is likely that the proportion of participants using these mobile devices will only increase. One potential contribution of timing variance is that the Mac computers we used had current macOS versions installed, which could have a negative impact compared to Windows devices. This is because Apple optimises their current operating systems to their most recent hardware. However, using an out-of-date operating system could also have had a negative impact on timing. As mentioned above, we had opted to not alter our set-ups where possible, but readers may like to consider any possible impact of OS on their results when recruiting participants. In any case, the relatively small invariance of devices reported here is good news for researchers, as the devices are often the variable that they are least able to control. This bodes well for the current state of online research.

At the time of testing, we used the most recent versions. Since then, Gorilla, jsPsych, Lab.js, and PsychoPy have all received updates. According to the developers of PsychoPy, their latest version has substantially improved timing (Bridges et al., 2020). Fortunately for the research community, software is often dynamic and constantly improved in regard to how it deals with presentation and data recording. Unfortunately, this means that any data reported in a paper will almost certainly reflect an older version of a software by the time of publication. All packages assessed here will likely have improved timing at some point in the future, so we encourage users who really need acute timing accuracy to gather external chronometrics themselves—as others also suggest (Bridges et al., 2020).

In our findings, particularly noteworthy are the larger delays (compared to lab-based software set-ups) in the recording of response times, which on average lag 80 ms, and extend to 100 ms on some set-ups. These results show larger delays and variance than recent results from Bridges et al. (2020), who limit the hardware elements of the sample to two devices with the same screen and low-latency button box. The authors also use different versions of software— older versions of jsPsych (v6.0 vs v6.0.5) and Lab,js (v2.4.4 vs v19.1.0) and a newer version of PsychoPy (v2020.1 vs v3.1.5). Lastly, for display duration, Bridges et al. (2020) use a smaller number of trials (1000 vs 4350) and a single duration (200 ms vs 16.66–500 ms). These differences are potential reasons for the different data we report. Researchers should keep these instances of larger delays in mind when conducting reaction-time-sensitive studies, by ensuring relative RTs are used (Pronk et al., 2019; Bridges et al., 2020). When timing sensitivity is crucial, we recommend employing within-participant designs where possible to avoid having to make comparisons between participants with different devices, operating systems, and browsers. Additionally, limiting participants to one browser could remove further noise. Limiting participants’ devices and browsers can be done programmatically in all tested platforms, and via a graphical user interface in Gorilla.

There were several extreme values reported in the results for the visual duration assessment, which we reported for completeness. These are a total of 22 trials out of 104,880 trials (.021%). They range from around 90 ms to 265 ms, and the causes are difficult to elucidate (even reproducing them, as a rare event, would be difficult). It is unlikely such outliers will impact researchers’ data at the group level, as these errors will appear roughly every 5000 trials. To put this into context, an example study showing 100 trials per participant would have one of these errors every 50 participants. No such extreme deviations appeared to happen in the reaction time data.

The accuracy and precision differences between set-ups are relatively small, and for most researchers the guiding factor for platform selection should be individual preference and ease of use. For those interested in particularly niche and time-sensitive tasks, platform selection strongly depends on the intended design and sample.

There is a difference in timing accuracy and precision between the presentation of stimuli and reaction times (not formally tested, but observable in Figs. 3 and 7). It is often the case that a task which demands an accurate and precise reaction time metric also requires reasonable display metrics.

The impact of timing error also changes depending on required display duration or magnitude of RT differences expected. For example, for those interested in presenting stimuli for a small number of frames or a single frame, a dropped frame may lead to a stimulus not being presented at all, or an over-presentation of one frame could double the display time, whereas for a user presenting stimuli for seconds, these same display errors would matter much less. When we think about RT error, the variance should be put into the context of human variance—often this is in the scale of hundreds of milliseconds, so variability of 10–30 milliseconds is unlikely to obscure clear differences between conditions (Damian, 2010). Researchers should take the time to look at expected RT magnitudes and use short display durations, and consider whether they need to take steps to improve timing performance, or whether any tool will provide good enough accuracy and precision.

In terms of informing researchers, this is among the most comprehensive assessments of timing accuracy across different platforms, browsers, and devices that have been carried out, in addition to Bridges et al. (2020). In general, our results indicate that no online platforms should necessarily be avoided, and that their timing characteristics are suitable for many types of research. Readers should avoid drawing strong conclusions from comparisons, as platforms, browsers, and operating systems evolve rapidly. We suggest researchers keep up to date with new releases of software, as timing could change substantially in the future.

## General limitations

Only a limited number of computers were used to collect the data presented here. As outlined in our results, participants in online experiments use a wide variety of software. In addition, their hardware will vary substantially, and each home computer will be equipped with its own unique ecosystem of software that can potentially interfere with timing accuracy.

Three of the authors (AAI, NH, and JKE) are employed by Cauldron, which operates the Gorilla experiment platform.

### Conclusions

Whilst offering larger sample sizes, web experimentation introduces variation in participants’ geographical locations and computer set-ups. We show that the accuracy and precision of display and response timing is not always consistent across different devices, operating systems, and experiment platforms, with no single platform standing out as the best. Our results also suggest that MTurk and Prolific participants are predominantly European and American, and that the best combination of browser and device (Chrome and Windows) is also the most common in use. Researchers who are keen to conduct online studies that include experiments for which timing is crucial would be wise to scrutinise the complex interactions between platforms, operating systems, and browsers, and opt for within-participant designs, or potentially consider restricting participants’ set-ups.

#### Open Practices Statement

This study was not pre-registered, as this was not appropriate given the lack of hypothesis testing reported. The data for RT and VDD assessments, in addition to the HTML for jsPsych, Lab.js, and PsychoJS, are available on the Open Science Framework at https://osf.io/rn9zd. The data from the user survey are not provided, as this is confidential user data from Gorilla.sc, containing partial personally identifiable information, and users have not consented to individual data-sharing.

#### Authors note

AAI, NH, and JKE are employed by Cauldron, which operates the Gorilla experiment platform. AAI’s PhD is funded by the Templeton World Charity Foundation (TWCF no. 0159) and the Medical Research Council.

## Data Availability

The data for RT and VDD assessments are available on the Open Science Framework at https://osf.io/rn9zd. The data from the participant analysis are not provided, as this is confidential user data from Gorilla.sc, containing partial personally identifiable information, and users have not consented to individual data-sharing.
